# “When you have a high life, and you like sex, you will be afraid”: a qualitative evaluation of adolescents’ decision to test for HIV in Zambia and Kenya using the health belief model

**DOI:** 10.1186/s12889-021-10391-x

**Published:** 2021-02-25

**Authors:** Leila Katirayi, Job Akuno, Bright Kulukulu, Rose Masaba

**Affiliations:** 1 Research Department, Elizabeth Glaser Pediatric AIDS Foundation, Washington, D.C USA; 2 Elizabeth Glaser Pediatric AIDS Foundation, Nairobi, Kenya; 3 Elizabeth Glaser Pediatric AIDS Foundation, Lusaka, Zambia

**Keywords:** HIV, Adolescents, Africa, HIV testing, Fear, Stigma, Peer influence, ART

## Abstract

**Background:**

HIV testing among adolescents is significantly lower than among adults and many adolescents living with HIV do not know their status. Adolescent perceptions of HIV testing are poorly understood and may negatively affect testing uptake. Using a qualitative design, this study sought to explore perceptions about HIV testing and treatment among adolescents living with HIV and adolescents of unknown HIV status in Lusaka, Zambia and Kenya.

**Methods:**

Study participants were adolescents aged 15–19 years old. The adolescents living with HIV were recruited from HIV support groups at health facilities. Adolescents of unknown HIV status were recruited from existing adolescent groups within the community. In both Zambia and Kenya, four focus group discussions (FGDs) were conducted with adolescents living with HIV and four FGDs were conducted with adolescents whose HIV status was unknown, for a total of 16 FDGs. FGDs consisted of 6–12 participants, a moderator, and a note-taker. FGDs were audio-recorded, transcribed, and translated into transcripts. Transcripts were coded in the qualitative analysis software program MAXQDA v. 12. Data reduction and summary tables were generated to help identify themes across the two study population groups. Data were interpreted within the health belief model.

**Results:**

Adolescents discussed the challenges of facing a positive HIV test result, including fear of a positive result and need to change their lifestyle, fear of social isolation, and perception of the lost opportunity to achieve future dreams. Most adolescents of unknown status were not as aware of the benefits of learning their HIV status, nor were they aware of the ability to live a long and healthy life on ART. HIV-positive adolescents reported that the messages targeted towards adolescents focus on the need to remain HIV-negative, as opposed to the benefits of knowing one’s status. Adolescents described age and requirements for parental permission as a significant limitation in their ability to access HIV testing.

**Conclusions:**

Adolescents require more information about the benefits of testing early and the ability to live a long and healthy life on ART. Educating adolescents that HIV testing is a normative behavior among their peers could strengthen HIV testing among adolescents.

**Supplementary Information:**

The online version contains supplementary material available at 10.1186/s12889-021-10391-x.

## Background

Adolescents and youth make up an increasing portion of people living with HIV (PLHIV). In 2019, an estimated 460,000 young people aged 10–24 years were newly infected with HIV worldwide [1]. Adolescent girls and young women (AGYW) remain disproportionally vulnerable and affected by HIV, accounting for 71% of new infections in young people in sub-Saharan Africa in 2018 [2]. Adolescents face unique barriers along the HIV cascade of testing, care, and treatment. Compared to adults, adolescents have lower rates of HIV testing, poorer ART access and coverage, higher loss to follow-up, poorer adherence, and lower rates of viral suppression [3, 4]. High HIV mortality continues to occur among adolescents further highlighting their vulnerability. AIDS is the leading cause of death among adolescents in Africa and the second leading cause globally [5].

### Adolescent uptake of HIV testing and counseling

Testing and linkage to care account for the largest drop off in the continuum of care among adolescents living with HIV (ALHIV) [6]. A significant proportion of ALHIV do not know their HIV status. Population-based surveys from 2015 to 2017 in selected countries in eastern and southern Africa revealed that only 46% of young people aged 15–24 were aware of their HIV status [7]. In 2019, only 27% of girls and 16% of boys aged 15–19 years were tested for HIV over a 12-month period and received their results in Eastern and Southern Africa [1]. Low levels of HIV testing access and uptake by adolescents is leading to late diagnosis and late entry into care and treatment [8].

Documented factors associated with the uptake of HIV testing among adolescents include having a higher level of education, being sexually active, over age 15 years, and having high HIV knowledge [9, 10]. While some adolescents may be very knowledgeable about HIV testing and counseling, many still choose not to test for HIV. One study from Tanzania found that despite high knowledge about HIV testing services (HTS), uptake of HTS among secondary students was very low [11].

Differences based on sex and gender also impact uptake of HIV testing. AGYW who become pregnant interface with antenatal care (ANC) services at health facilities, which presents them with an opportunity for HIV testing. Receiving ANC has been associated with increased uptake of HIV testing [12]. In comparison, men have lower rates of HIV testing than women [13].

Previous literature exploring what discourages adolescents from receiving an HIV test found correlations with fear of a positive result, age, education, and stigma. Fear is one of the most documented barriers to testing, including concerns of stigma and discrimination around testing; fear of a positive test and the impact of a positive diagnosis; ostracization by peers, partners, and family; and being associated with promiscuous behavior [14–16]. Fears can be born out of insufficient or incorrect information and knowledge, which that can be exacerbated by peers, partners, and family [16].

Moreover, other factors that act as barriers for adolescent HIV testing uptake include poor accessibility to HTS due to transport, cost, service hours of health facilities, and negative attitudes or behaviors from health providers, including being judgmental and not maintaining confidentiality [17, 18]. On a structural level, consent requirements pose barriers for adolescents in accessing testing services. Parents’ fear of HIV-related stigma and consequences and a desire to protect their child can result in a lack of parental support for HIV testing [19]. Cultural taboos may also deter parents from discussing sexuality and HIV with their adolescents [20]. The literature reveals the impact parental consent requirements have on those under 18 years in deterring use of sexual and reproductive health services, especially among adolescent girls [7, 20].

This study aimed to explore the factors that influence adolescents’ decisions to test for HIV and to understand why many adolescents choose not to test. This paper explores the perspective of adolescents who have tested HIV-positive and those who have not tested for HIV.

### Theoretical application

Study results have been interpreted within the health belief model (HBM), a framework developed in the 1950s by social psychologists Irwin M. Rosenstock, Godfrey M. Hochbaum, S. Stephen Kegeles, and Howard Leventhal [21]. The model identifies four determinants of behavior change: perceived threat (influenced by perceived susceptibility and severity), cues to action, self-efficacy, and perceived benefits vs. barriers. An individual’s beliefs about these factors determine their likelihood to engage in health-promoting behavior. The premise of the model is that one must believe the benefits of the recommended action outweigh the potential consequences [22]. Modifying variables, primarily demographic and psychosocial (such as age, gender, education, socio-economic status, etc.), influence these perceptions.

### Country-specific context

Zambia is one of the countries hardest hit by the HIV epidemic. In 2019, the HIV prevalence among adults (aged 15–49 years) was 11.5% [23]. HIV prevalence among the youth ages 15–24 years is estimated to be 5.6% for women and 1.8% for men [24]. The 2018 Zambia Demographic Health Survey reported that approximately 59% of female adolescents and 46% of male adolescents aged 15–19 know their HIV status [24].

The Republic of Kenya is another country significantly affected by the AIDS epidemic. In 2019, with a population exceeding 51 million, Kenya had approximately 1.5 million PLHIV [25]. In Kenya, 51% of new infections occurred among adolescents and young people in 2015 [26]. HIV prevalence among young adults aged 15–24 is estimated to be 1.3% (males) and 2.6% among (females). Annual new HIV infections for adolescents aged 10–19 is estimated to be 8200 [27]. Only 23.5% of adolescents aged 15–19 know their HIV status [28].

## Methods

### Study design

This qualitative study used focus group discussions (FGDs) to explore perceptions about HIV testing and treatment among ALHIV and adolescents of unknown HIV status in Lusaka, Zambia and Kisumu, Kenya.

### Study population

Data were collected from ALHIV and adolescents of unknown HIV status who were 15–19 years old. ALHIV were eligible if they knew their HIV status and were utilizing support services at the health facility at the time of study recruitment. Adolescents of unknown HIV status were eligible if they were in the age range of 15–19 years. These adolescents were recruited from existing adolescent groups within the community, such as soccer clubs. This group was meant to represent typical adolescents and we did not seek out adolescents with a specific HIV status. The study team did ask if these adolescents had tested for HIV, but did not ask result of the test. Since the study team was unaware of the HIV status of these adolescents, this group is referred to as ‘adolescents of unknown HIV status.’

### Site selection

Kisumu and Lusaka were selected because they were urban locations where adolescent services were being provided by the Elizabeth Glaser Pediatric AIDS Foundation (EGPAF). ALHIV were recruited from existing facility-based support groups at two selected ‘model’ sites in Kisumu and Lusaka. Model sites had monthly psychosocial support groups (PSSGs), linkages to schools to support students living with HIV, adolescent defaulter tracking, and youth-friendly services. The youth-friendly services included a dedicated adolescent provider and a flexible schedule for adolescents to access services. In addition, two sites in need of adolescent program reinforcement were selected. Adolescents of unknown HIV status were recruited from existing community organizations in Kisumu and Lusaka that had regularly occurring adolescent meeting groups. These groups were selected based on the ease of access to adolescents (frequency of meetings, number of participants, etc.).

### Data collection

At each of the four selected model health facilities (two per country), one FGD was conducted with HIV-positive adolescents, for a total of four FGDs with ALHIV. Similarly, one FGD was conducted with adolescents of unknown HIV status from each of the four community groups identified, for a total of four FGDs with adolescents of unknown status. Data were collected in November 2016.

To recruit ALHIV from existing support groups and community groups, the study developed a recruitment script for leaders to introduce the study and its purpose and invite the adolescents to return on a specific day/time to attend the FGD. FGDs took place at the location where the groups regularly met. If more than the maximum number of eligible participants arrived at a FGD, the research assistants (RAs) were instructed to organize a second FGD on another day, however, this situation did not occur.

The FGDs were led by RAs who were hired and trained by EGPAF. Each FGD included 6–12 study participants and was moderated by one RA with another RA taking notes. FGDs guides were semi-structured and explored topics such as where adolescents received information about HIV testing, who influenced the adolescents’ decision to test, what encouraged testing, and questions about what services and facility attributes made a facility ‘adolescent friendly.’ For more information, see supplementary file 1- FGD guide- Adolescents with unknown HIV status and supplementary file 2- FGD guide- HIV-positive adolescents. FGDs in Kenya were conducted in Dholuo, English, and Kiswahili. FGDs in Zambia were conducted in English, Nyanja, and Bemba. All FGDs were audio-recorded.

Adolescents 18 years and older provided written informed consent before joining the study. Adolescents under the age of 18 were asked to return with a caregiver to provide written consent, while the adolescent signed an assent form. In Kenya, emancipated minors, defined as those who are married, head of the household, pregnant, or have children were able to provide written consent on their own behalf according to national policy [29].

### Analysis

FGD audio-recordings were simultaneously translated and transcribed into Word documents. Transcripts were reviewed by the study team to generate the initial code list, using an inductive approach. Transcripts were coded by two RAs, under the supervision of the qualitative lead using MAXQDA v.12. The code list was modified as emergent themes arose during the coding process. Once coding was complete, code reports (in Word format) were generated by country and study group (Zambia vs. Kenya, and HIV-positive vs. unknown status). The HBM was used to interpret the findings after inductive coding was complete. Data were analyzed by study group, comparing the responses of ALHIV to the responses of the adolescents of unknown status. In addition, data from Zambia and Kenya were compared for similarities and differences. Data reduction and summary matrices were generated to aid in the identification of overall themes and key findings.

## Results

The mean age in both countries and both population groups was approximately 17 years old (Tables 1 and 2). In both Zambia and Kenya, there were fewer males in the HIV-positive group (less than 50%) and a greater proportion of males in the adolescents of unknown status group (close to 60%). In both countries, and across both study population groups, the majority of participants were enrolled in school. For those who were living with HIV, most were on ART and had disclosed their status to family members. For those of unknown HIV status, the majority (81% in Zambia and 95% in Kenya) had been offered an HIV test and most had accepted the test (78% in Zambia and 97% in Kenya). These testing acceptance rates were higher than expected when compared to national HIV testing rates of adolescents in Zambia and Kenya. The demographic data, including HIV testing history, was gathered individually before the FGDs were conducted.

**Table 1:** Adolescent Groups from Lusaka, Zambia

Characteristic	Living with HIV (***n*** = 53)	Unknown HIV Status (***n*** = 32)
**Age (years)**		
Mean [95% confidence interval]	17.5 [17.2, 17.9]	17.7 [17.3, 18.1]
**Gender**		
Male	25 (47.2%)	13 (59.3%)
**Education**		
Not in School	6 (11.3%)	1 (3.1%)
In School	30 (56.6%)	17 (53.1%)
Graduated	17 (32.1%)	14 (43.8%)
**Living Situation**		
Live at home	49 (92.5%)	32 (100%)
Other	4 (7.5%)	
**On ART**		
Yes	45 (84.9%)	–
No	8 (15.1%)	–
**Disclosed to**^a^		
Family	40 (75.5%)	–
Friends	15 (28.3%)	–
Community	1 (1.9%)	–
Church	1 (1.9%)	–
None	3 (5.7%)	
**Offered an HIV Test**		
Yes	–	26 (81.3%)
No	–	6 (18.7%)
**Accepted an HIV Test**		
Yes	–	25 (96.2%)
No	–	1 (4%)

^a^Some adolescents reported disclosing to multiple categories

**Table 2:** Adolescent Groups from Kisumu, Kenya

Characteristic	Living with HIV (***n*** = 35)	Unknown HIV Status (***n*** = 38)
**Age (years)**		
Mean [95% confidence interval]	17.1 [16.5, 17.1]	16.8 [16.3, 17.2]
**Gender**		
Male	13 (37.1%)	22 (57.9%)
**Education**		
Not in school	8 (22.9%)	–
In school	25 (71.4%)	33 (86.8%)
Graduated	2 (5.7%)	5 (13.2%)
**Living Situation**		
Live at home	29 (82.9%)	37 (97.4%)
Live at school	5 (14.3%)	–
Other	1 (2.9%)	1 (2.6%)
**On ART**		
Yes	34 (97.1%)	–
No	1 (2.9%)	–
**Disclosed to**		
Family	34 (97.1%)	–
School	1 (2.9%)	–
**Offered an HIV Test**		
Yes	–	36 (94.7%)
No	–	2 (5.3%)
**Accepted an HIV Test**^a^		
Yes	–	35 (97.2%)
No	–	1 (2.8%)

^a^Missing data from two participants

The results below have been organized by the HBM constructs: perceived susceptibility, perceived severity, perceived threat of disease, cues to action, self-efficacy, likelihood of action, and perceived benefits vs. barriers to behavioral change.

### Perceived susceptibility

Adolescents were well aware of their potential risk of contracting HIV. Many adolescents who believed that they had a high probability of testing HIV-positive were unmotivated to seek HTS out of fear of an HIV-positive test result.

*Mostly when you have a high life, and you like sex, you will be afraid because you know what you do because you may assume that when you go, you might find things are bad. So, it becomes a barrier. Therefore, you opt not to know. (Zambian adolescent, unknown status).*

Fewer adolescents reported the opposite perspective, that their risky sexual behavior encouraged them to learn their status. These individuals reported preferring peace of mind over the fear of a HIV-positive outcome.

Sometimes because of sleeping a lot with women, you start thinking maybe I have contracted the virus. That’s what influences people to go and test. (Zambian adolescent, unknown status)

### Perceived severity

Perceptions about an HIV-positive status being equal to a death sentence was expressed by many adolescents and discouraged them from testing for HIV. This opinion was expressed more by the adolescents of unknown HIV status.

If I tell my friend I want to go and get tested, she will discourage me by telling me, when you go and know your status you will die, taking medication every day is not easy, so she will discourage me, when I put all that in mind I will not go get a test (Kenyan adolescent, unknown status)

Some adolescents made statements about potential suicidal behavior, if one was found to be HIV-positive.

My friend says that, ‘when [I] am found with HIV, I can kill myself.’ (Kenyan adolescent, unknown status)

### Perceived threat of disease

Many adolescents discussed a fear of ‘missing out’ on life’s opportunities as being one of their concerns. Adolescents also expressed their desire to further their education, establish rewarding careers, and raise families. However, many adolescents believed that being HIV-positive would prevent them from fulfilling these dreams, so they were reluctant to learn their HIV status.

When you say future, I ask myself [about] adulthood. What if I don’t even make it there, you know— a career that I will start for 5 years doctoring? What if I just end at 3 years and that is my time? (Zambian adolescent, unknown status)

Many adolescents feared the social consequences of being HIV-positive. Fear of losing critical social relationships with an HIV-positive status discouraged adolescents from testing.

Friends won’t be playing with you the way they used to when you find out your status. You will be isolating yourself from them. I am not the same as they are, so it will be much more difficult for them, so like you get scared to go test. (Zambian adolescent, HIV-positive status)

Similarly, adolescents felt that it would be difficult to find love with an HIV-positive status.

Other people were also saying that if you are taking drugs, people will not love you because you have HIV. (Kenyan adolescent, HIV-positive status)

Adolescents also worried about the reaction of their families and potential consequences. They feared an HIV-positive status could lead to rejection by their family.

If parents discourage you, then you yourself will be like no [I] am dead. Love comes from parents, if parents don’t love you, then no one else will do that. (Zambian adolescent, HIV-positive status)

Adolescents were also concerned about the financial implications of an HIV-positive status and putting additional financial pressure on their family.

Already, your family finds it hard to make ends meet. A person who’s HIV positive [has] to be on a balance diet, meaning it will have pressure on them cause they now have to add up some money for you to have a good balanced diet, hence you making your already poor family become more poor. (Zambian adolescent, unknown status)

### Cues to action

Seeing parents taking daily medications prompted adolescents to want to learn their own HIV-status.

*I wanted to know my status, since I was very sick, and I was sure my parents were taking drugs*. *(Kenyan adolescent, HIV-positive status)*

Some adolescents reported unknown illness prompted them to get tested, although they had no initial suspicion of being HIV-positive.

I was sick once but after several medications, it just got worse. So, a friend whom I was staying with told me to go get tested. If it’s malaria, I get medication, but unfortunately, I was found HIV-positive. (Kenyan adolescent, HIV-positive status)

Knowledge among peers about the symptoms of being HIV-positive encouraged adolescents to test. One adolescent described her symptoms and experience as an HIV-positive person to her friends, which encouraged them to get tested. *You tell them the effects of HIV, like when you are positive, you frequently get ill, so you need to get tested to ascertain whether you are positive or negative. (Kenyan adolescent, HIV-positive status)*

### Self-efficacy

Adolescents described their age and requirements for parental permission as a major influencer in their ability to access HIV testing. An individual’s young age was reported to be a limiting factor to access HIV testing. This was due to the requirement in both Zambia and Kenya that individuals must be 16 years of age or older or be accompanied by a guardian or caregiver to receive an HIV test at the heath facility.

*The first time I went to the clinic, individually, to go and test. They told, me ‘how old are you?’ I said 16. They said, ‘you are too young to get tested, so you should just go back, maybe come with your mother.’ You see, so they discouraged me, saying I was too young to get tested. (Zambian adolescent, unknown status).*

Some adolescents were hesitant to receive a HIV test in front of their parents, as it was an admission of being sexually active.

*Since you have to get the consent from the parents, he or she will be there while testing. If you are found positive and you know your behaviors, you will have a lot of problems with your parents*. *(Kenyan adolescent, unknown status)*

Adolescents discussed challenges accessing HTS with each other. Becoming aware of each other’s negative experiences may have discouraged other adolescents from attempting to access HIV testing.

[I] am a young person, aged 15, and when I go to the clinic to access maybe HTS, the first thing that I will incur from there is the facial expression by the caregiver. As a young person, I will be discouraged to go for HTS, and when am discouraged when I come back, I will tell my friend as well, so these are some of the messages that we receive from our fellow adolescents, those that have done HTS before at the clinic. They tell us their experiences, which often times are negative. (Zambian adolescent, unknown status)

While some adolescents reported healthcare workers (HCWs) turning them away due to their young age, many adolescents also described positive interactions with HCWs, including providing positive counseling messages about living a long and healthy life on ART. This result was more commonly reported by ALHIV.

Some adolescents, especially HIV-positive adolescents, reported being comfortable with HCWs and getting reassurance that they would be able to continue with life once they learned their status. It was important for them to have the ability to discuss their concerns because it helped to mitigate their fears.

I think before I knew that I was HIV-positive, I came and brought my sister, and I wasn’t tested, so they encouraged me to test that even if you are found negative or positive, it doesn’t mean it’s the end of your life, so I was tested. I was found to be positive. My mind was very confused, but in care of the counselors they helped me through their discussions and through the way they told me like it’s not like your life is over, but it is the beginning of life. (Zambian adolescent, HIV-positive status)

Additional services developed for youths, such as the Youth Friendly Corners (YFCs) offered a safe environment for adolescents to express their concerns and connect with other ALHIV.

In schools they also do mention youth friendly corners, so in case you not comfortable to go to the guidance counseling office where ever you go for YFC in the community, at a clinic, you can go there you are insured you will find young people to find whatever you want to ask. (Zambian adolescent, unknown status)

### Likelihood of action

Participants, especially those with unknown HIV status, were lacking information about the benefits of knowing one’s HIV status.

The power of the unknown, you know, not knowing what you [are] going to find, this is what actually scares a lot of youths nowadays. If they find me with HIV, what happens next? (Zambian adolescent, unknown status)

Some participants discussed that most of the messages targeting adolescents were about remaining HIV-negative, while very few messages focused on the ability to live a long and healthy life on ART.

*The information has an impact, but there is only one issue. You find that you focus mainly to those people who are negative, but there is less focus to those who are positive. Often times, you talk condoms whenever you are having sex, but you don’t talk about treatment especially treatment and care. Treatment, care, and support to people who [have] HIV and AIDS. So, the information is there, but is only one-sided to those people who are negative. Those people who are positive are excluded.* (*Zambian adolescent, unknown status)*

Some participants felt that additional information about HIV testing and treatment would encourage adolescents to test and learn their status.

*I think if adolescents know or are given information on HIV services, I think for me they can utilize information. Because looking at I myself, personally, I never used to go for HTS until I knew the importance of HTS that’s when I started going for HTS. (Zambian adolescent, unknown status).*

### Perceived benefits vs. barriers to behavioral change

Many adolescents discussed that their peers would prefer to not learn their HIV status to avoid any required lifestyle changes.

The perception most of the people have in the community, even at school, is they decide not to do an HIV test because they are living a good life, so if they go for the possibility that [I] am HIV-positive, it will be affecting their lives. So, they will decide not to go and test for HIV. Instead they just live the life that they are living. (Zambian adolescent, unknown status)

## Discussion

According to the HBM, the recommended action (HIV testing), will occur when the benefits are believed to outweigh the consequences. Adolescents discussed at length the disadvantages to knowing one’s HIV status, yet minimal information was shared about the benefits of knowing one’s HIV status.

### Perceived consequences of testing for HIV

The first component of the HBM is perceived threat, which is influenced by perceived susceptibility and severity. Some research has found that adolescents underestimate their own risk of being HIV-positive, which decreases their chances of getting tested for HIV [30]. Other research, including results from this study, illustrate that adolescents are very aware of their HIV risk, and consequently do not want to get tested for HIV [16, 31]. In our study, participants had a strong sense of perceived threat and severity of an HIV-positive diagnosis, which appeared to be strongly related to fear of social consequences. While many participants discussed that HIV meant ‘death,’ the interpretation of ‘death’ appeared to be as much social as physical. Previous studies support the findings that adolescents fear social rejection by their peers, family members, and community [16, 32]. In South Africa, study participants reported that the stress of knowing one is HIV-positive is more likely to negatively affect one than AIDS itself [33].

Adolescence is a unique time, when adolescents begin to develop their own identities, much of which is shaped by their peers and social interactions. During this time, the weight of social acceptance is high and the threat of social rejection is severe. Peer networks are a critical support system for adolescents and fear of losing this support system discourages many adolescents from testing for HIV [33].

In our study, adolescents faced many challenges to accessing HTS, partly due to the minimum age requirement of 16 years. Participants reported being uncomfortable to receive an HIV test in front of their parents. Adolescents also reported HCWs turning them away or making comments that they should return for testing when they are older. Sharing stories of being turned away resulted in other adolescents becoming discouraged to attempt to access testing.

### Perceived benefits of testing for HIV

While adolescents were well aware of the potential consequences of an HIV-positive status, they were less aware of the benefits of knowing one’s status. The main benefit of HIV testing mentioned by the study participants was peace of mind. Adolescents may have seen their parents taking drugs for treatment, experienced illness themselves, or had concerns based on their own sexual activity and this prompted them to want to test and know their HIV status.

Most adolescents of unknown HIV status did not discuss the ability to live a long and healthy life on ART, nor the importance of starting treatment early. While ALHIV had a stronger understanding of the benefits of testing and starting treatment early, many reported that they received this information after testing for HIV.

### Changing the perception that the consequences of learning one’s HIV status outweighs the benefits

Participants reported that HIV messages targeted towards adolescents only reinforce the message to remain HIV-negative, which is interpreted as there being ‘no other option’ than to remain negative. HIV-positive adolescents discussed the positive impact of HCWs sharing messages about the ability to live a long and healthy life on ART and that such messages should be shared earlier and with the adolescent population in general, not just to those who seek health services. Providing positive messages about the ability to live a long and healthy life could help reduce the fear around a HIV-positive test result, potentially encouraging more adolescents to test. Further research is needed to evaluate what messages are being shared with adolescents and what messaging, in terms of content and wording, would be most effective in reaching adolescents.

Adolescents, especially males, lack a consistent source of accurate information about HIV and reproductive health. Females will likely be screened for HIV testing when they become pregnant and access ANC, but adolescent males do not have a similar opportunity. A study in Malawi found that men who were aged 20 and older were more likely to have been tested for HIV compared to adolescent men in the age range of 15–19 years [34]. This study recommended designing programs, policies, and strategies to target adolescent men. HIV testing rates among men remain lower than women across sub-Saharan Africa [35]. The challenge of men accepting HIV testing and treatment services is well documented [36, 37]. A study in Tanzania found that knowledge of HIV testing procedures and peer encouragement were instrumental in encouraging men to test for HIV [38]. In this study, men reported that their peers encouraged them to test for HIV because they themselves had been tested, providing them with motivation and information. Increasing men’s knowledge of HIV testing from a young age may help shift HIV testing norms among men.

Throughout the FGDs, participants discussed the challenges of adolescents testing for HIV. However, the demographic data, which was captured individually with participants before the FGDs, indicated that the majority of adolescents of unknown HIV status had previously accepted HIV testing. FGDs are designed to explore the social norms of a selected group of people, not the individual experience. This data highlights a divergence between the perceived norm and actual norms regarding adolescents testing for HIV. A cross-sectional, population-based study in rural Uganda found that the majority of participants misperceived HIV testing uptake as not normative in their community, when it actually was normative [39]. This study also found that those who thought that testing was not normative were much less likely to have tested for HIV.

Minimal research has explored adolescents’ perceptions of HIV testing norms within their communities. While this sample size is small, it does highlight the need for further exploration of adolescents’ perceptions of HIV testing among their peers. Perceptions of whether a behavior is normative or not, influences the uptake of the behavior [40]. According to the social norms approach, interventions which aim to correct misperceptions by exposing actual norms will benefit individuals, by reducing problem behaviors and increasing healthy behaviors [40]. Educating adolescents that HIV testing is a normative behavior among their peers could strengthen HIV testing among adolescents.

### Study limitations

Since urban sites were selected, this study did not capture the experiences and perspectives of adolescents living in more rural and remote locations. Study participants in urban areas may have greater knowledge of and access to HTS. In addition, only one urban area was selected per country, providing a limited perspective of the urban environment. Participating ALHIV were selected from facilities with ‘youth-friendly’ services, which may have resulted in adolescents having more positive experiences at the health facilities. The study populations were also allowed to self-select, which may have introduced some level of bias, with those who may have had particularly ‘good’ or ‘bad’ experiences wanting to share their experience.

## Conclusion

Due to fear of social exclusion and insufficient information about HIV and ART, adolescents had a heightened sense of ‘consequence’ about testing positively, in part due to their age and the influential role of their peers. According to the HBM, the decision to change one’s behavior is a result of the perceived benefits outweighing the consequences. The results from this study highlight that adolescents generally perceive the consequences of an HIV-positive result to outweigh the potential benefit of knowing one’s HIV status. To shift this balance and encourage adolescents to test for HIV, adolescents require more information about the benefits of testing early and the ability to live a long and healthy life on ART. While there are programs targeting AGYW, who are most vulnerable, and young pregnant women in ANC, there is a need for targeted programs and strategies encouraging HIV testing among adolescent males.

Finally, our study showed a disconnect between adolescents’ perceived HIV testing norms and actual norms. Educating adolescents that HIV testing is normative among peers could strengthen HTS uptake.

## Supplementary Information

**Additional file 1:.** Supplementary material- FGD guide- Adolescents with unknown HIV status- data collection tool used for focus group discussions with adolescents whose HIV status is unknown to research team

**Additional file 2:.** Supplementary material-FGD guide- HIV-positive adolescents- data collection tool used for focus group discussions with adolescents living with HIV and accessing care.

## Data Availability

Due to concerns about participants’ privacy, this dataset is not available publicly.

## Abbreviations

AIDS: Acquired immunodeficiency syndrome
AGYM: Adolescent girls and young women
ALHIV: Adolescents living with HIV
ANC: Antenatal care
ART: Antiretroviral therapy
EGPAF: Elizabeth glaser pediatric AIDS foundation
FGD: Focus group discussion
HBM: Health belief model
HCW: Healthcare worker
HIV: Human immunodeficiency virus
HTS: HIV testing services
PLHIV: People living with HIV
PSSG: Psychosocial support group
RA: Research assistant
YFC: Youth friendly corner
