# Supplementary material for: “When you have a high life, and you like sex, you will be afraid”: a qualitative evaluation of adolescents’ decision to test for HIV in Zambia and Kenya using the health belief model
Source: BMC Public Health. 2021 Feb 25;21:398. doi: 10.1186/s12889-021-10391-x (PMC7905429; doi:10.1186/s12889-021-10391-x)
Supplement: Supplementary file 1 — Additional file 1:. Supplementary material- FGD guide- Adolescents with unknown HIV status- data collection tool used for focus group discussions with adolescents whose HIV status is unknown to research team [file 12889_2021_10391_MOESM1_ESM.docx]

**Understanding Adolescent HIV Services in Lusaka, Zambia: An Evaluation of Acceptability and Appropriateness**

**Focus Group Discussion Guide- Adolescents of unknown HIV status**

**Version 1.0**

| Date of the FGD | __ __ / __ __ / __ __ __ __ (dd-mm-yyyy) |
| --- | --- |
| Site Number | __________________________________ |
| Research Assistant FGD Moderator | __________________________________ |
| Research Assistant FGD Note Taker | __________________________________ |

FGD Number _____

Number of Participants __ __

Start time __ __: __ __

**Introduction:**

Introduce the moderator and note-taker. Explain that we are here to learn more about adolescents’ perceptions of health services at the facility so that we can improve adolescent care.

Assign participant numbers to be referred to throughout the FGD. Explain that this helps protect their privacy and makes it easier for the note taker to capture what was said.

**Perspectives about HIV in General**

1. What is said among adolescents about HIV?
2. Do adolescents feel safe discussing HIV and asking questions?
   (Probe: at school, at the health facility, with caregivers, etc.).

**Community Perspectives about Adolescents Accessing HIV Services**

1. At school, what is said at school about HIV services for adolescents?
   1. What messages are shared?
   2. What is said about adolescents testing for HIV?
2. In the community, what is said about adolescents using HIV services?
3. Are parents/caregivers supportive of adolescents testing for HIV? If yes, how? If no, why not?
4. Are siblings and other family members supportive? If yes, how? If no, why not?
5. What is said in the religious institutions about adolescents testing for HIV?
6. Who shares these messages?
7. How likely are adolescents to listen to these messages?

**Reaching Adolescents**

1. Do adolescents have enough information about HIV in general?
   (Probe: Knowledge about HIV transmission, protection methods, medication for HIV, living with HIV, etc.)
2. What resources in the community (that currently exist) share information about HIV with adolescents? (For example, youth clubs, girls groups, school groups for adolescents living with HIV, church groups, health clinics, peer facilitators, etc.)
3. What are the best ways to share information about HIV services with adolescents?
   (Probe to get many suggestions, including social media ideas, word of mouth, pamphlets, posters, radio, etc.)
   1. Where do youth spend time?
4. Do you know if adolescents are getting tested in your community?
5. (Probe: Where do they go to be tested?)
6. If adolescents know about HIV services for adolescents, do you think they will utilize the facility? (Probe to understand why or why not)
7. What can be done within the community to encourage more adolescents to access HIV services?

**Accessing the Health Facility**

1. How easy is it to access a health facility?
   (Probe to understand if it easy to reach the facility, facility hours are acceptable, if there are stigma/social factors to dissuade them from visiting the health facility)
2. Do you think adolescents under 16 experience challenges receiving a HIV test? (Remind them that parents have to consent for adolescents under 16)
3. We want to make the health facility as friendly/welcoming for adolescents as possible to receive HIV testing and health services. Please tell me your ideas about what would make the health facility friendly and welcoming for adolescents. (Let them speak first, if there is silence give them small hints, like other adolescents assisting at the facility, a special corner just for adolescents, etc.)

**Adolescent Services**

*HIV Testing & Service Knowledge*

1. What have you heard about the confidentiality of receiving a HIV test?
2. For those who have been tested for HIV or heard stories about their friends being tested, please tell us what you have heard about adolescents’ experiences getting tested for HIV.
3. What are the barriers that adolescents might face when getting tested for HIV?
4. What encourages adolescents to get tested for HIV? (Let them speak first, if they are quiet suggest items like receiving flyers about testing, having tested offered at locations that adolescents frequently attend, such as schools, etc.)
5. If you were to find out that you are HIV-positive, who is the first person that you would disclose your status to?
6. Once an adolescent tests positive for HIV, what services have you heard are offered? What do you think happens to that adolescent?

*Sexual & Reproductive Health*

1. For those who have accessed a health facility or have friends that have accessed a facility, what have you heard about the information, counseling support, and services provided to adolescents? (Probe on the following topics: partner relationships, protection (condoms), family planning, etc.?)
   1. What additional information should be provided?

End time: __ __: __ __
