# Supplementary material for: “When you have a high life, and you like sex, you will be afraid”: a qualitative evaluation of adolescents’ decision to test for HIV in Zambia and Kenya using the health belief model
Source: BMC Public Health. 2021 Feb 25;21:398. doi: 10.1186/s12889-021-10391-x (PMC7905429; doi:10.1186/s12889-021-10391-x)
Supplement: Supplementary file 2 — Additional file 2:. Supplementary material-FGD guide- HIV-positive adolescents- data collection tool used for focus group discussions with adolescents living with HIV and accessing care. [file 12889_2021_10391_MOESM2_ESM.docx]

**Understanding Adolescent HIV Services in Lusaka, Zambia: An Evaluation of Acceptability and Appropriateness**

**Focus Group Discussion Guide – HIV-positive Adolescents**

**Version 1.0**

| Date of the FGD | __ __ / __ __ / __ __ __ __ (dd-mm-yyyy) |
| --- | --- |
| Site Number | __________________________________ |
| Research Assistant FGD Moderator | __________________________________ |
| Research Assistant FGD Note taker | __________________________________ |

FGD Number _____

Number of Participants __ __

Start time __ __: __ __

**Introduction:**

Introduce the moderator and note-taker. Explain that we are here to learn more about adolescents’ perceptions of health services at the facility so that we can improve adolescent care.

Assign participant numbers to be referred to throughout the FGD. Explain that this helps protect their privacy and makes it easier for the note taker to capture what was said.

**Accessing the Health Facility**

1. How easy is it to access services at this health facility? (Probe to understand if the location is easily accessible, facility hours are acceptable, organization of services at the facility, time to receive services, comfortable waiting area, and physical space is welcoming, etc.)
2. How do you feel about the space in which adolescent services are provided in? (Probe to understand how they feel about safety and privacy of the space in which the adolescent services are provided)
3. Do you feel you spend too little or too much time on clinic day?

**HCWs Providing Services**

1. How do you feel about the HCWs providing HIV services?
   1. Probe to understand if they feel comfortable, safe, respected, supported, etc. Ask the youths to provide you with examples of what HCWs have done to make them feel this way.
   2. Do you have preferences to receive care from a HCW of your own gender?
   3. What could the HCWS do differently to improve how they provide HIV services for youths?
2. Who are you comfortable talking with? Peers counselors, nurses, doctors, pharmacy staff? (Probe- who do you speak with when visiting the facility? Would you like to spend more or less time with any of these groups?)

**HIV Testing Services**

1. What prompted you to seek out HIV services?
2. What encouraged you to get tested for HIV?
   a. Did you experience any challenges with getting tested? (Please describe)
3. What influences you and your friends’ decision to test for HIV or not? (Probe to understand what could be done to encourage youths to test)
4. How did you hear about where to get tested for HIV?

**Adolescent HIV Services**

1. Is there a youth-friendly corner at this facility? Are there adolescents here to greet you?
   (Probe- What would make this youth-friendly corner even more friendly and welcoming for you? What would an ideal youth-friendly corner look like?)
2. How do you feel about the HIV services offered at this facility?
3. What are some of the services that you really like at this facility?
4. What are some of the services that you wish they would change? (Probe to understand how they could be improved)
5. What are other services you think youth would like to receive that are not currently offered?

*Transition to Adult Care*

1. How is the clinic preparing you to move over to adult care?
   (Probe: Are you satisfied with these services?)
2. What are some of the concerns you have about becoming an adult while living with HIV?
   (Probe: Future relationships, staying alive, being different, having children, etc.
   1. What can be done to better assist you and help you plan for your future?

*Disclosure*

1. What kinds of support have you received from the facility to help you disclose to your peers, partners and family members? (Probe- What additional support would you like to receive for disclosure?)
2. What has helped you to disclose? What strategies or tactics?

*Sexual & Reproductive Health*

1. What kinds of information and counseling support services have you received regarding relationships, protection (condoms), family planning, etc.? (Probe- What additional services would you like to receive?)
2. How has living with HIV affected your ability to be in an intimate relationship?

**Adherence**

1. When adolescents initiate treatment, is the information provided adequate? (Probe- Is information shared regarding why treatment is important, the dangers of missing a dose, etc.)
2. Once an adolescent knows that they are HIV-positive, what encourages them to start ART or not?
3. What helps you to adhere?
   1. Does anybody help remind you take your meds or encourage you to stay on your meds?
   2. What additional support would help you adhere?
   3. What other techniques have you used? (i.e. putting medications in different bottles to disguise contents, etc.)
4. What are some of the barriers that prevent you from adhering?
   1. What are the reasons you might skip your medications? (Probe- Have you experienced any challenges to getting your medications? Have you experienced any side effects?)
5. How does the facility contact you before your appointment? If you miss an appointment, how does the facility follow-up with you to remind you to return to the facility? (Probe- How would you like to be contacted?)

**Community Perspective**

1. For those who are in school, what is said at school about HIV services for adolescents?
   1. What messages are shared at school about HIV?
   2. What are the attitudes among your peers about adolescents using HIV services?
2. What is said where you stay (home or boarding school) about using HIV services?
3. Are your parents/caregivers supportive of you using these HIV services? If yes, how? If no, why not?
4. Are siblings/housemates and other family members supportive? If yes, how? If no, why not?
5. What is said at church about youths using HIV services?
6. Who shares these messages?
7. Do youths listen to these messages?

**Reaching Adolescent**

1. Do adolescents have enough information about HIV?
   (Probe: Knowledge about HIV transmission, protection methods, medication for HIV, living with HIV, etc.)
2. What resources in the community (that currently exist) share information about HIV with adolescents? (For example, youth clubs, girls groups, school groups for adolescents living with HIV, church groups, etc.)
3. What are the best ways to share information about HIV services with adolescents?
   (Probe to get many suggestions, including social media ideas, word of mouth, pamphlets, posters, radio, etc.)
   1. Where do youth spend time?
4. If adolescents know about HIV services for adolescents, do you think they will utilize the facility? (Probe to understand why or why not).
5. What can be done within the community to encourage more adolescents to access HIV services?

End time __ __: __ __
